# Investigating the use of generative AI policies among ASPPH member schools and programs of public health

**DOI:** 10.3389/fpubh.2026.1796810

**Published:** 2026-04-08

**Authors:** Ashish Joshi, Brian McGoldrick, Nidhi Mittal, Shongkour Roy, Zebunnesa Zeba, Michael Arthur Ofori, Stella Dockery, Niharika Jha, John Auerbach, Jean Cadet, Jessica Kruger, Robert Leeman, Kerry Mitchell, Guillaume Molter, Andrew Naidech, Elahe Nezami, Sylvester Orimaye, Courtney Queen, Umair Shah, Abdul Shaikh, Daniel Smith, Melissa Tracy, Edward Trapido, Jorg Westermann, Andrew Wiss, Jonathan Zaccarini, Tim E. Leshan, Linda A. Alexander, Jamie M. Atchison, Eduardo Ruiz, Laura Magana

**Affiliations:** 1 School of Public Health, University of Memphis, Memphis, TN, United States; 2 ICF, Fairfax, VA, United States; 3 School of Population Health, Andrews University, Berrien Springs, MI, United States; 4 Teaching Innovation and Excellence, School of Public Health and Health Professions, University at Buffalo, New York, NY, United States; 5 Public Health and Health Sciences, Bouvé College of Health Sciences, Northeastern University, Boston, MA, United States; 6 Department of Public Health and Preventive Medicine, St. George’s University, St. George’s, Grenada; 7 Administrative and Web Services, Harvard T.H. Chan School of Public Health, Boston, MA, United States; 8 Program in Public Health, Northwestern University Feinberg School of Medicine, Chicago, IL, United States; 9 Department of Health Sciences, University of Miami, Miami, FL, United States; 10 College of Global Population Health at UHSP in St. Louis, St. Louis, MO, United States; 11 Julia Jones Matthews School of Population and Public Health, Texas Tech University Health Sciences Center, Lubbock, TX, United States; 12 Phamily, Cincinnati, OH, United States; 13 Global Leader for Population Health, Amazon Web Services (AWS), Seattle, WA, United States; 14 College of Graduate Health Studies, A.T. Still University, Mesa, AZ, United States; 15 Department of Epidemiology and Biostatistics, University at Albany College of Integrated Health Sciences, Albany, NY, United States; 16 Louisiana State University Health Sciences Center School of Public Health, New Orleans, LA, United States; 17 School of Health Sciences, Walden University College of Health Sciences and Public Policy, Minneapolis, MN, United States; 18 Milken Institute School of Public Health, George Washington University, Washington, DC, United States; 19 School of Public Health, Columbia University Mailman, New York, NY, United States; 20 Association of Schools and Programs of Public Health, Washington, DC, United States

**Keywords:** AI, AI use, ASPPH, generative AI, policy, public health

## Abstract

**Background:**

The integration of generative artificial intelligence (AI) into the operational and curricular foundations of higher education is a dynamically evolving issue, particularly in public health programs looking to ethically embrace the ever-growing potential of AI in health research and education. Despite a collective priority across the United States to effectively adopt generative AI, few works have characterized the scope and rigor of existing guidance.

**Methods:**

This study employs a mixed-method design to characterize AI use guidance offered by member schools of the American Schools and Programs of Public Health. Guidance documents are screened for relevance and accessibility, with the remaining documents being classified as guidelines or policies. The determined policies are analyzed for content via descriptive analysis, content analysis, TF-IDF analysis, and thematic analysis.

**Results:**

Out of a possible 155 schools, 18 schools were determined to have a generative AI use policy. TF-IDF analysis identified recurring terms of importance throughout the corpus of policy documents. Content and Thematic Analysis identified areas of focus and themes shared throughout the corpus of documents. The resulting triangulated data are used to identify strengths and shortcomings in the extant policies with suggestions for further development.

**Conclusion:**

The results of the mixed-method design indicate that extant generative AI guidance in schools and programs of public health in the United States is in its relative infancy. Shared themes and areas of focus show a predominant focus on the intersection of academic integrity and AI use among students and faculty. Additionally, data privacy and security, research ethics, and other areas are currently under consideration. This study demonstrates that AI use governance is growing but narrow in scope. Further development in the areas of pedagogy, legal guidance, and ethical use frameworks are needed in order to meet the growing demand to integrate generative AI into US higher education.

## 1 Introduction

The rapid advancement of artificial intelligence (AI), particularly generative artificial intelligence (GenAI), has introduced new capabilities that allow machines to generate text, images, code, and analytical outputs with minimal human input. These technologies are increasingly being adopted across sectors, including higher education, where they are influencing teaching, learning, research, and administrative practices (1, 2). The emergence of large language models and similar tools has prompted universities to reconsider how digital technologies interact with traditional academic norms such as authorship, originality, and assessment integrity (3, 4).

The diffusion of generative AI in educational environments has occurred rapidly, often outpacing institutional governance structures. Technology adoption theories such as the Technology Acceptance Model (TAM) and Innovation Diffusion Theory (IDT) suggest that the uptake of emerging technologies is influenced by perceived usefulness, ease of use, and institutional context (5). Within higher education, students and instructors have increasingly incorporated generative AI tools into coursework, research, and communication tasks, creating both opportunities for innovation and challenges related to academic oversight and responsible use (1, 6). These developments have prompted universities to begin developing institutional responses intended to guide or regulate the use of AI within academic settings.

Institutional responses to emerging technologies often take the form of policies, guidelines, or broader governance mechanisms, each reflecting different levels of formality and enforcement. In general, institutional policies refer to formally adopted rules that establish enforceable standards for acceptable behavior, while guidelines typically provide recommended practices that inform but do not mandate institutional conduct. Governance frameworks encompass the broader organizational structures and processes through which institutions oversee technology adoption, risk management, and accountability (7, 8). Clarifying these distinctions is important because universities have adopted a wide range of responses to generative AI that vary in scope, institutional authority, and implementation.

Existing literature indicates that institutional responses to generative AI in higher education remain heterogeneous and rapidly evolving. Early institutional guidance frequently focused on academic integrity concerns, particularly the potential for AI-assisted plagiarism or unauthorized authorship (4, 9). Subsequent scholarship has highlighted the need to move beyond restrictive approaches toward more comprehensive frameworks that incorporate AI literacy, responsible use, and pedagogical innovation (2, 10). For example, Moorhouse et al. (11) analyzed guidelines from leading global universities and found that fewer than half had developed explicit policies addressing generative AI in assessment practices, with most emphasizing the redesign of assignments and the promotion of AI literacy. Similarly, An et al. (12) identified substantial variation across higher education institutions in how generative AI is addressed within teaching, research, and administrative contexts. These findings suggest that institutional approaches to AI governance remain fragmented and institution-specific.

A growing body of scholarship has also examined the broader ethical and governance implications of AI in educational systems. International organizations and policy bodies have emphasized that the integration of AI into education should be guided by principles such as transparency, accountability, equity, and human oversight (13, 14). Ethical frameworks for AI in education highlight concerns related to algorithmic bias, privacy protection, data security, and the potential impact of automated systems on educational equity (8). Scholars have further argued that generative AI challenges long-standing assumptions about originality and individual authorship in academic work, requiring institutions to reconsider assessment practices and learning outcomes (3). These discussions provide normative guidance for responsible AI use but offer limited empirical insight into how institutions operationalize these principles through formal policy documents.

Empirical studies examining institutional AI governance remain relatively limited. Existing research has largely focused on general university policies or educator perceptions of generative AI adoption (6, 12, 15). For example, McDonald et al. (15) analyzed institutional AI policies across universities and found that early responses often focused narrowly on misconduct detection rather than broader pedagogical integration. Similarly, Lyu et al. (6) reported mixed perceptions among instructors regarding the benefits and risks of generative AI, highlighting ongoing uncertainty regarding appropriate institutional responses. These studies demonstrate that institutional strategies for managing AI technologies are still developing and often vary across institutions and disciplinary contexts.

Within this evolving policy landscape, relatively little research has examined how AI governance is being addressed in public health education. Schools and programs of public health operate within interdisciplinary academic environments that emphasize ethical responsibility, community engagement, and the protection of population health. Consequently, institutional decisions regarding emerging technologies may have implications not only for classroom practices but also for professional norms and workforce development within the public health field.

The Association of Schools and Programs of Public Health (ASPPH) represents a network of accredited schools and programs that collectively shape public health education and training. ASPPH has recently initiated efforts to explore the responsible and ethical use of AI within public health education, research, and practice, reflecting growing recognition of the technology’s potential impact on the field (16). Despite these developments, there is limited empirical evidence describing how ASPPH member institutions currently address generative AI through formal institutional policies or guidance.

Given the rapid diffusion of generative AI tools and the evolving institutional responses to them, systematic examination of existing policy approaches within public health education can provide insight into how institutions are interpreting and managing this technological shift. Rather than evaluating the effectiveness of these policies, the present study focuses on documenting and characterizing their presence, structure, and content.

The purpose of this study is therefore to systematically investigate and characterize how schools and programs of public health within the U.S. are developing and articulating policies and guidelines on the responsible use of GenAI. Specifically, this research focuses on member institutions of the ASPPH, which represent a diverse network of public health education providers across the country and abroad. The objectives for this study are threefold:

1. Identify which ASPPH member schools and programs have publicly available institutional guidance on AI use.
2. Identify which member schools have guidance that rises to the level of a policy.
3. Characterize and describe key components of the officially adopted, institution-wide policies.

This research seeks to present a clearer understanding of how public health programs are navigating the dual imperatives of innovation and responsibility. It offers evidence to guide future policy development, highlights areas where additional institutional support may be needed, and lays the groundwork for more consistent standards across the ASPPH network.

## 2 Materials and methods

### 2.1 Document gathering and data extraction

ASPPH’s “Academic Program Finder” was used to create our sample. Three research team members extracted data from October 2024 to January 2025 from each ASPPH member’s website. Documents containing AI policies/guidelines were then downloaded from 153 members’ respective websites. One of the member schools, the Colorado School of Public Health, is a collective of three universities, this resulted in an initial collection of 155 documents. Each ASPPH member website was reviewed to determine if there were any references to the AI Policies/Guidelines at the school or university level.

To ensure consistency and reproducibility of the document search process, each institutional website was systematically reviewed using a standardized procedure. Reviewers first examined the primary institutional pages associated with the school or program of public health, including sections commonly labeled “Policies,” “Academic Integrity,” “Teaching and Learning,” “Student Resources,” or “Information Technology.” If no AI-related documents were located through navigation, reviewers used the institutional website search function with the following keywords: “artificial intelligence,” “AI,” “generative AI,” “GenAI,” “ChatGPT,” and “AI policy.”

If an institutional website referenced AI policies hosted at the broader university level (e.g., institutional IT policies or academic integrity guidelines), those documents were also retrieved and included in the dataset. When webpages contained references to AI policies but the full documents were not accessible, the document was coded as unavailable and excluded from analysis. All identified documents were downloaded in PDF format and archived by the research team at the time of collection to ensure consistency in the analyzed versions despite potential future website updates.

### 2.2 Variable extraction

The following variables were extracted from the information on all AI policies and guidelines across the various academic schools and programs of public health in the US: school name, city, state, geographical region, type of university (public vs. private), audience of the respective policy or guideline and AI tools used. Additionally, we gathered information pertaining to the relevant documents’ discussions of AI applications (teaching, learning, research, etc.) and key considerations relating to AI use from the 128 available policies and guidelines.

A standardized data extraction template was developed prior to data collection to ensure consistent documentation of institutional characteristics and document attributes across reviewers. Each reviewer independently entered extracted variables into the shared data extraction sheet, after which entries were cross-checked by another member of the research team to ensure accuracy and completeness.

### 2.3 AI policy/guideline screening

Three research team members took a consensus coding approach and reviewed each AI policy/guideline document to determine whether it could be considered a policy. Formally designating guidance as policy ensures consistently enforceable standards and reduces ambiguity for students, faculty, and staff regarding appropriate AI use. These determinations were based on policy-identifying criteria established by the University of Wisconsin-Madison (UWM) Policy Library (17). The UWM Policy Library framework was selected because it provides a clearly defined and operationalized set of criteria distinguishing institutional policies from guidance documents within higher education governance systems. Although developed within a specific institutional context, the framework offers a practical structure for identifying enforceable policy characteristics that can be applied to policy analysis in other academic settings.

Specifically, each reviewer looked for language that: “states an institutional position”, “mandates, specifies or prohibits behavior”, “has broad applicability”, “contains enforceable, non-negotiable elements”, and “articulates both the ‘what’ and ‘why’ of the required action”. It should be noted that the UWM Policy Library also stipulates that a policy should “change infrequently.” However, given the novelty and ongoing development of AI regulation at every level, the research team agreed that this prerequisite could not be attributed to any AI guidance accurately and decided to omit it as a criterion. The research team decided purposively *a priori* that if four of the five characteristics were present, the document would be coded as a policy. If a document, regardless of title, did not meet this threshold, it would be coded as a guideline. If an individual reviewer felt that a document should be considered a policy despite the lack of four of the five criteria, they were given the opportunity to discuss it during the team review.

Prior to the full screening process, the three reviewers conducted a calibration exercise using a small subset of documents to ensure consistent interpretation of the classification criteria. During this stage, reviewers independently coded the documents and then compared results to clarify ambiguities in the criteria and harmonize coding decisions. The research team focused their screening on the content of the document, resulting in multiple documents being coded as “policies” even if they were not titled explicitly as such. Following the individual coding of each document, the research team convened and discussed individual findings before deciding via consensus what the group’s determination would be. Disagreements between reviewers were resolved through structured discussion during team meetings. If consensus could not be reached immediately, the document was re-reviewed collectively with reference to the predefined criteria until agreement was achieved.

### 2.4 Analyses

Multiple analytical techniques were used to address different components of the study objectives. Krippendorf’s Alpha (Kα) was employed to assess the agreement of the raters beyond chance and the inter-rater reliability of the rating scheme, given its suitability with multiple raters and rigor against missing and non-random data (18). The potential values range from −1 to 1, with an alpha of 1 indicating perfect agreement, an alpha of 0 indicating no agreement beyond what would occur by chance, and an alpha below 0 indicating systemic disagreement (19). In this study, each rater independently provided ratings. The rated data were input into R and used to calculate KA using Hayes’ KA macro (20). Additionally, the percentage of agreement was calculated to indicate base agreement not accounting for chance.

Descriptive statistics were used to summarize institutional characteristics and the distribution of policies and guidelines across schools and programs of public health. Content analysis was used to categorize institutional documents based on predefined variables extracted during the data collection process (e.g., audience, AI tools referenced, and areas of application such as teaching, research, or learning). Thematic analysis was used to identify recurring themes within the documents coded as formal policies. Finally, Term Frequency (TF) and Inverse Document Frequency (IDF) text mining was used as an exploratory technique to identify frequently occurring terms and patterns within the policy corpus, providing an additional quantitative description of the language used across documents.

TF, IDF, and TF-IDF are three commonly used statistics in data mining, text classification, and natural language processing research. TF values are representative of a word’s relative frequency in a single document, IDF values represent the rarity or commonness of a word across a collection of documents, and TF-IDF values are the products of TF and IDF values that represent a term’s importance in a single document in the context of a corpus (collection) of similar documents. In this study, they were used collectively to determine the importance of common words in each document and across the 18 documents collectively. Prior to analysis, each document was cleaned and stemmed. Additionally, all mentions of “artificial intelligence” were changed to “AI”, all periods were manually removed from “A.I.”, and “generative AI” was changed to “genai”. Analysis was performed using the R statistical software (20). These modifications were applied solely for the purposes of the TF–IDF analysis to standardize variations in terminology referring to the same concept. The original wording of the documents was preserved for all qualitative analyses, including thematic coding and policy classification, to ensure that interpretive accuracy and contextual meaning were maintained.

Once the document screening was complete, four research team members characterized and summarized key components of the documents coded as policies. Following this, the resultant data was organized into a single document, which was then used as the basis for the development of a thematic codebook. The codebook was developed using an inductive thematic analysis approach. Initially, two members of the research team independently reviewed the policy documents to identify recurring topics related to AI governance, institutional expectations, and academic applications of AI. Preliminary codes were generated during this stage and discussed among the research team to consolidate overlapping concepts and refine code definitions. The finalized codebook included clearly defined thematic categories with descriptions and inclusion criteria to guide consistent application during coding.

After the codebook was finalized, four research team members applied the thematic codes to the documents coded as policies. Coding was conducted iteratively, with periodic team discussions to clarify interpretations and ensure consistent application of the coding framework. When discrepancies occurred, they were resolved through group discussion and consensus review of the relevant document sections. Themes were considered sufficiently developed when no new thematic categories emerged during the iterative coding process across the policy documents. This process allowed the research team to identify recurring patterns in institutional AI governance language while ensuring that the thematic categories adequately represented the content of the analyzed documents.

## 3 Results

### 3.1 Variable extraction

Descriptive data was extracted from the 128 publicly available member policies and guidelines governing AI use. Full results delineated by policy, guideline, and total can be found in Supplementary material.

### 3.2 Document screening

**Figure 1:**
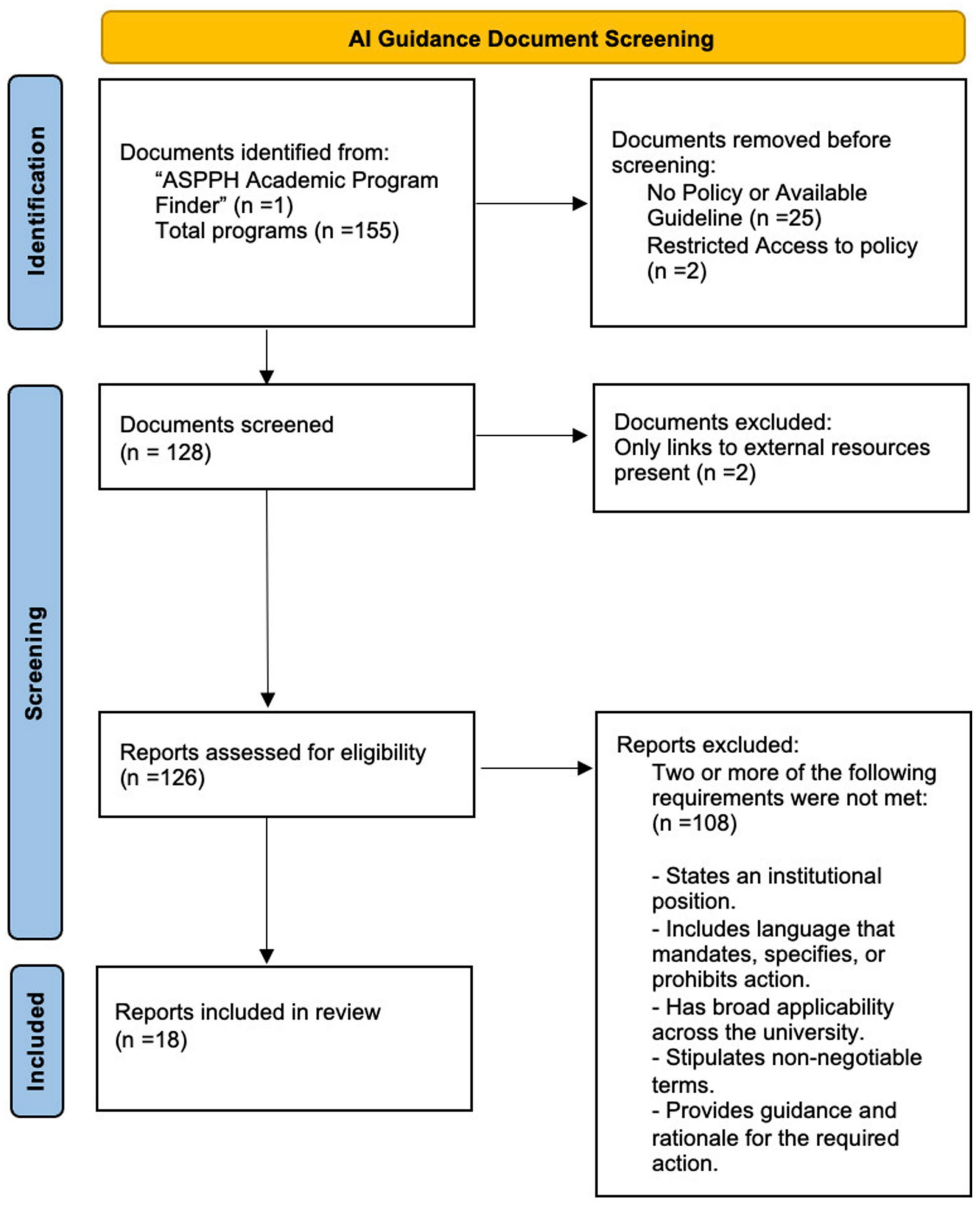
Flow chart diagram for identifying AI policy documents across ASPPH member schools and programs of public health.

An initial screening of 155 documents across the ASPPH schools and programs showed that 23 of these documents had no guidance on AI use and hence were excluded. Documents from two institutions were not publicly available and hence were excluded from the analysis. Following the review of 128 documents, 108 were excluded from the final analysis as they were missing two or more of the characteristics listed in the inclusion criteria. Two institutions had only external links but not their own guidelines or policies. This resulted in a final group of 18 AI policies. A flowchart document showing the screening process is shown in Figure 1.

### 3.3 Inter-rater reliability

Krippendorf’s Alpha was generated to assess the reliability of the coding scheme and the extent of agreement among the raters beyond chance. The resulting Kα coefficient was 0.514 (Kα = 0.514, [0.310, 0.676]). The percentage of agreement between the three raters was 82%.

### 3.4 TF-IDF analysis

TF-IDF analysis was conducted on the five most common terms across all 18 documents. The five most common words across all 18 documents (post-stemming and cleaning) were “use”, “student”, “tools”, “genai”, and “course”. Total TF-IDF values can be found in Table 1.

**Table 1:** TF-IDF analysis.

Doc	TF					IDF					TF-IDF				
	Use	Student	Tools	GenAI	Course	Use	Student	Tools	GenAI	Course	Use	Student	Tools	GenAI	Course
1	0.0472	0.0236	0.0198	0.0028	0.0038	1	1	1	1.02	1.042	0.0472	0.0236	0.0198	0.0029	0.004
2	0.0324	0.0162	0.0378	0.0162	0.0108	1	1	1	1.02	1.042	0.0324	0.0162	0.0378	0.0165	0.0113
3	0.0492	0.0055	0.0246	0.0082	0	1	1	1	1.02	1.042	0.0492	0.0055	0.0246	0.0084	0
4	0.0451	0.0088	0.0442	0	0.0044	1	1	1	1.02	1.042	0.0451	0.0088	0.0442	0	0.0046
5	0.0304	0.0118	0.0374	0.0346	0.0042	1	1	1	1.02	1.042	0.0304	0.0118	0.0374	0.0353	0.0044
6	0.0345	0.0292	0.0424	0.061	0.0584	1	1	1	1.02	1.042	0.0345	0.0292	0.0424	0.0622	0.0609
7	0.0255	0.0185	0.0046	0.0486	0.0023	1	1	1	1.02	1.042	0.0255	0.0185	0.0046	0.0498	0.0024
8	0.0428	0.035	0.0195	0.0233	0.035	1	1	1	1.02	1.042	0.0428	0.035	0.0195	0.0048	0.0365
9	0.0167	0.0369	0.0047	0.0012	0.0202	1	1	1	1.02	1.042	0.0167	0.0369	0.0047	0.0012	0.021
10	0.0656	0.0063	0.0188	0.0656	0.0438	1	1	1	1.02	1.042	0.0656	0.0063	0.0188	0.0669	0.0456
11	0.0655	0.0087	0.0218	0.0393	0.0087	1	1	1	1.02	1.042	0.0655	0.0087	0.0218	0.0401	0.0083
12	0.0772	0.0112	0.0244	0.002	0.0213	1	1	1	1.02	1.042	0.0772	0.0112	0.0244	0.002	0.0222
13	0.0419	0.0576	0.0314	0.0262	0.0105	1	1	1	1.02	1.042	0.0419	0.0576	0.0314	0.0267	0.0109
14	0.0255	0.0255	0.0036	0.0291	0	1	1	1	1.02	1.042	0.0255	0.0255	0.0036	0.0297	0
15	0.0419	0.0209	0.043	0.0452	0.0143	1	1	1	1.02	1.042	0.0419	0.0209	0.043	0.0461	0.0149
16	0.0483	0.0181	0.0498	0.0227	0.0227	1	1	1	1.02	1.042	0.0482	0.0181	0.0498	0.0232	0.0237
17	0.0079	0.0394	0.0026	0.0105	0.0105	1	1	1	1.02	1.042	0.0079	0.0394	0.0026	0.0107	0.0109
18	0.0717	0.0169	0.0313	0.0169	0.0143	1	1	1	1.02	1.042	0.0717	0.0169	0.0313	0.0146	0.0149

### 3.5 Content analysis

Content analysis of the 18 policies revealed 7 areas of focus and frequency counts on how many of the policies covered said area. These areas include “AI Integrity/ Misconduct Guidance”, “Usage Policy/ Pedagogical Guidance”, “Legal Compliance”, “Ethical Considerations”, “Data Privacy/Security Guidelines”, “Software Access Guidance”, and “AI Detection Software/ Disciplinary Guidance.”

“AI Integrity/Misconduct Guidance” was defined as language that specifies how AI use may violate academic integrity and/or be considered academic misconduct. Fourteen out of eighteen universities reviewed included academic integrity guidelines related to AI use in their institutional policies. Most of these policies instructed the students to seek prior permission from the faculty for using AI during the course. Of the 18 policies examined, two policies acknowledged the growing significance of AI in education but left it to individual instructors to decide its use within the course.

“Usage Policy/ Pedagogical Guidance” was defined as language that dictates how faculty may/ should integrate AI tools into their curriculum. Fifteen of the eighteen institutions provided guidance related to AI use in the classroom. Most policies provide strata of AI use restriction for instructors to choose from for their course policy. These generally include complete prohibition, restricted use, and unrestricted use. Some universities allow instructors to create their own AI use statement instead of following a standard one. To facilitate this, these institutions further provide internal and external resources to help instructors design their own course policy.

“Legal Compliance” was defined as guidance that directs how faculty, staff, and students should use AI tools in accordance with external policies/ laws (i.e., federal, state, research agency, etc.). Four out of 18 policies have guidance requiring compliance with legal frameworks, including the Health Insurance Portability and Accountability Act (HIPAA), the Family Educational Rights and Privacy Act (FERPA), and other state regulations. One of the institutional policies emphasized complying with the rules laid by federal funding agencies like NIH. Another institution prohibits the use of AI applications like Deep Seek AI on government devices. One of the policies elaborated on the risks associated with AI use, i.e., infringement of intellectual property rights and data breaches. This institutional policy further warns the users that AI-generated content might lead to bias or discrimination, which would be considered a violation of the institution’s values and legal responsibilities.

“Ethical Considerations” were defined as guidance that suggests/dictates how stakeholders should use AI to align with ethical standards and/ or broadly cautions against known harmful results of AI use (i.e., biased information, misinformation, hallucinations, etc.). Among the 18 institutional AI policies reviewed, five explicitly mention ethical concerns related to the use of generative AI. These policies cite algorithmic bias, inaccuracies in output, and potential infringement of intellectual property rights as risks associated with the use of AI tools for work. Some of the institutions even forbid users from inputting any confidential or personally identifiable information into AI platforms, considering data protection issues. Additionally, some policies assign the responsibility of using any AI-generated output in their work to the user.

“Data Privacy/ Security Guidelines” were defined as guidance that mandates what information can or cannot be entered in LLM’s or other AI tools. Nine of the eighteen reviewed universities discussed data privacy and security in their generative AI policies. The methods of governance also differed greatly, ranging from elaborate multi-level systems to simple measures limiting sensitive data utilization, which suggests that institutions are yet to come up with consistent policies of integrating AI innovations and security requirements. In response to the provisions of FERPA, HIPAA and GDPR, universities often forbid the entry of sensitive information such as student identifiable information, unpublished research materials, grants, and confidential information into GenAI models. In one institution, a 4-tier data classification framework (Public, Internal, Confidential/Sensitive, Highly Restricted) was created, whereas another institution provided strict faculty policies that prevented the entry of any identifiable information. The institutions offer holistic guidelines covering the issues of information security, data privacy, copyright, and academic integrity. They take into consideration that AI inputs can be exposed, and outputs can be biased or false. Many higher educational institutions have formal approvals, such as departmental review boards and governance systems. Transparency was one of the key values, as disclosure of the AI usage and data limitations had to be documented.

“Software Access Guidance” was defined as language that outlines processes for acquiring/ approving new AI tools. Seven of the eighteen universities have clear instructions on the use and acquisition of generative AI tools. In these cases, approval for use is often required before faculty, staff, or students can use certain generative AI software. In addition to the regulation of AI tools themselves, some institutions govern the use and availability of AI-detection software. This manifests as blanket bans of tools like Turnitin as the sole determinant of AI misuse, protocols pertaining to which scenarios detection tools can be used, and informed consent procedures for students whose faculty have opted to use AI detection tools.

“AI Detection Software/ Disciplinary Guidance” was defined as language that offers or mandates how AI detection software may be used and/or dictates how disciplinary action involving AI use is carried out. Four of the eighteen universities have a defined policy regarding the use of AI detection tools and how to deal with AI misuse. Multiple schools have explicit centralized academic misconduct processes, meaning that detection of AI misuse by the faculty is not the sole determinant of misconduct. Conversely, one institution requires student consent should their faculty opt to use AI detection software. It is important to note that while every policy mentions AI in the context of academic misconduct, only these four contained explicit policies detailing disciplinary procedures. Frequency of each area can be found in Table 2.

**Table 2:** Content analysis.

Area of focus	Frequency	Percentage (*n* = 18)
Usage policy/ pedagogical guidance	15	83.3%
Academic integrity/ misconduct guidance	14	77.8%
Data privacy/ security guidelines	9	50%
Software access guidance	7	38.9%
Ethical considerations	5	27.8%
Legal policy compliance	4	22.2%
AI detection/ disciplinary action guidance	4	22.2%

### 3.6 Thematic analysis

We performed thematic analysis on the final 18 policies. In total, 6 themes, 10 subthemes, and 21 categories were developed. Definitions for each category and textual examples were documented as well. In total, our thematic analysis identified 6 themes, 10 subthemes, and 21 categories across the final 18 policies. These themes were further separated by general audience, with two themes primarily aligning with faculty responsibilities (“AI Use in the Classroom” and “Misconduct Involving AI Use”), one with student responsibilities (“AI Use and Academic Integrity”), one with staff/administrative responsibilities (“Sensitive Data Governance”), one with researcher responsibilities (“Ethical Implications of AI Use in the University Setting”), and one that is universal across all audiences (“Extra-Institutional Considerations”).

“AI Use in the Classroom” contained two subthemes with five categories: “Classroom Usage Policies” (“Tiered Usage Policies, Instructor-Driven Policies”) and “Instructor Responsibilities” (“Transparency”, ‘Practicality”, and “Privacy Risk Transparency”). This theme primarily concerns governing AI use in learning environments in addition to considerations for instructors developing or enforcing AI use policies.

“Misconduct Involving AI Use” contained two subthemes with four categories: “Disciplinary Action” (“Broad Disciplinary Guidelines”, “Established Processes”) and “AI Detection Software Use” (“Using AI Software to Determine AI Misuse”, “Policies Governing AI Detection Use)”. This theme concerns faculty enforcement of policies enforcing ethical and integrity-driven use of generative AI, in addition to stipulations on the use of AI detection software.

“AI Use and Academic Integrity” contained two subthemes and four categories focused on students as an audience: “AI Use as a Violation of Academic Integrity” (“Prohibition Sans Instructor Approval”, “Prohibition Sans Human Involvement”) and “Citation and Accountability” (“All AI Use Must be Cited”, “Student Accountability and Responsibility”). This theme primarily dealt with the broad governance of student AI use.

“Sensitive Data Governance” contains two subthemes and three categories with Staff/Administrators as an audience: “Data Security” (“Blanket Bans to Sensitive Data Input”, “Tiered Bans to Sensitive Data Input”) and “IT Management” (“Software Procurement”). This theme concerned preventing violations of data privacy and security via regulation of data entry into LLMs. Additionally, it addressed AI tool procurement and screening.

“Ethical Implications of AI Use in the University Setting” contained two themes, two subthemes, and two categories focused on researchers as an audience: Research and Intellectual Property Ethics (“Research Integrity”, “Intellectual Property Concerns”) and “Funding Agency Guidelines.” This theme focused on the intersection of research integrity and AI use as a complex and potentially problematic subject.

“Extra-Institutional Considerations” contained two subthemes and two categories with all stakeholders as an audience: “Aligning with Federal and State Law” and “Human-Centered Ethics” (“Onus of Responsibility on the Individual”, “Potential for Harm as a Result of AI Use”).

These themes characterize the corpus of analyzed policies but are present throughout the vast majority of the original 155 documents, even if the documents themselves did not display the necessary rigor to be considered a policy. Despite the current novelty of AI use and infancy of its regulation, these themes indicate current priorities in AI use guidance in higher education. The full codebook, with textual examples for each subtheme, can be found in Table 3.

**Table 3:** Thematic analysis.

Audience	Themes	Subthemes	Categories	Textual examples
Faculty	*AI Use in the Classroom*	*Classroom Usage Policies*	Tiered Usage Policies	Syracuse University: “… academic integrity violations for inappropriate use of artificial intelligence will not be investigated unless the course syllabus contains one of the three artificial intelligence statements provided below.”
			Instructor-Driven Policies	University of Pennsylvania: “Individual instructors determine their own policies related to generative AI. “
		*Instructor Responsibility*	Transparency	George Washington University: “If an instructor wishes to permit certain uses of GAI tools, such uses must be set forth explicitly in the course syllabus”
			Practicality	University of Nebraska:”… Given how easy it is to use A. I. and how difficult it is for instructors to detect, these policies will be difficult to enforce…”
			Privacy Risk Transparency	Mercer University:”…You may want to consider reminding your students to exercise caution when interacting with AI applications to avoid unintentionally sharing intellectual property, copyrighted materials, or confidential data.”
	*Misconduct Involving AI Use*	*Disciplinary Action*	Broad Disciplinary Guidelines	Baylor University: “AI policy violations will be addressed by the appropriate academic dean’s office and the Office of the Provost.”
			Established Processes	SUNY Upstate: *See MPH AI Policy under ‘Procedures’*
		*AI Detection Software Use*	Using AI Detection Software to Determine AI Misuse	SUNY Upstate: “The course instructor uses appropriate technology to determine likelihood and extent of AI generated content. “
			Policies Governing AI Detection Use	Syracuse University: “In order to comply with University policies and federal and state law…instructors who plan to use the software program Turnitin…are required to notify students in advance of their intent to do so.”
Students	*AI Use and Academic Integrity*	*AI Misuse as a Violation of Academic Integrity*	Prohibition Sans Instructor Approval	SUNY Upstate: “If not explicitly approved by the course instructor, using AI, generative or otherwise, to write or create any substantive part of any assignment – will be considered a form of plagiarism and will be considered a breach of academic integrity.”
			Prohibition Sans Human Involvement	Baylor University “… AI-generated results must undergo human review…
Students (cont.)	*AI Use and Academic Integrity (cont.)*	*Citation and Accountability*	All AI Use Must be Cited	University of Alabama at Birmingham: “Creative content originating from non-human origin (e.g., artificial intelligence) should be clearly attributed to the originating analytics program used.”
			Student Accountability and Responsibility	George Washington University: “Work submitted for evaluation is represented as the student’s own intellectual product”
Staff/Administrators	*Sensitive Data Governance*	*Data Security*	Blanket Bans to Sensitive Data Input	University of Alabama at Birmingham: “Do not upload Sensitive or Restricted/PHI data into generative AI systems.”
			Tiered Bans to Sensitive Data Input	East Carolina University: “For “Level 1-Public” data, any generative AI products or services are allowed. For levels 2, 3, and 4, protected institutional data…only Generative AI products or services listed on the Sensitive Data Storage and Transmission website are approved.”
		*IT Management*	Software Procurement	Baylor University: “Any new generative AI platforms and chatbots must undergo review and approval by Baylor ITS and the Office of the Provost.”
Researchers	*Ethical Implications of AI Use in the University Setting*	*Research and Intellectual Property Ethics*	Research Integrity	East Carolina University: “Faculty must also refer to the ECU Libraries website’s Generative AI in the Classroom & Research: Research Best Practices on the use of AI for research.”
			Intellectual Property Concerns	Columbia University: “…if Generative AI is given access to confidential information or trade secrets, the University may lose its intellectual property (IP) rights to that information and the information may be disclosed to unauthorized third parties …
	*Ethical Implications of AI Use in the University Setting (cont.)*	*Funding Agency Guidelines*		University of Alabama Birmingham: “Use of generative AI in the National Institutes of Health (NIH) peer review process is prohibited. Use of AI in the development of a grant proposal should be consistent with funding agency policy statement and otherwise validated, appropriately cited, and disclosed.”
All stakeholders	*Extra-Institutional Considerations*	*Aligning with Federal and State Law*		George Mason University: “Per Executive Order 46, no employee of any agency of the Commonwealth of Virginia shall download or use the DeepSeek AI application on any government-issued devices, including state-issued cell phones, laptops, or other devices capable of connecting to the internet.”
		*Human-Centered Ethics*	Onus of Responsibility on the Individual	Baylor University: “Users of AI-generated results bear full responsibility for ensuring compliance.”
All Stakeholders (cont.)			Potential for Harm as a Result of AI Use	University of Colorado: “Be vigilant about the presence of biases in work generated by AI/ML and strive to prevent dissemination of these biases.”

## 4 Discussion

This study characterized the institutional guidance on AI use provided by member schools of the Association of Schools and Programs of Public Health via descriptive analysis, text mining, TF-IDF analysis, content analysis, and thematic analysis. Similar to An et al., this study examined each policy and guideline in the context of all relevant stakeholders including students, faculty, staff, and researchers (12). The collective results of all analyses provide insight into the current state of AI governance among ASPPH member institutions and paths forward for development and innovation. It is important to note that many schools circulate AI policy internally and do not formally publish AI guidelines on their websites.

Although we demonstrate how current policies focus on academic integrity and ethical use, more schools are reviewing their programs to address AI integration in crucial career-building skills that will equip public health students for future career growth.

### 4.1 Guideline and policy descriptive analysis

The vast majority of the total ASPPH members’ guidance on AI usage mentions at least one piece of AI software by name, with only 11 total schools not mentioning a specific tool. ChatGPT is the most often-discussed tool, with 77.78% of member schools explicitly mentioning it. Other discussed tools include DALL-E (19.84%), Google Bard (19.84%), Google Gemini (15.87%), and Amazon Claude (15.87%), among others. These findings align with trends in college AI usage, as reports indicate that ChatGPT is the LLM of choice for many college students across the globe (21).

Most of the analyzed documents explicitly discussed applications of AI in at least one context of higher education. The most common applications discussed include teaching (89.96%) and learning (82.54%), with topics like work-related applications, research, content creation, data analysis, and others also being mentioned. This indicates a primary emphasis on guidance on AI use in the classroom setting. This aligns with other studies on AI use in university settings that report governance of AI use in the classroom as the predominant focus (11, 12).

Many of the analyzed documents provided commentary and discussion on AI use that went beyond the scope of governance of use. Topics of discussion include the reliability of AI software (5.56%), bias concerns (6.35%), lack of accuracy (7.94%), copyright concerns (2.38%), privacy (3.17%) and confidentiality (12.7%) concerns, transparency of governance around AI use (9.52%), and faculty facilitation of AI usage (53.97%). These considerations were generally presented as rationales for a particular policy or guideline or as an item of concern for a faculty member building their individual AI guidance principles.

In terms of the intended audience of each school’s respective guidance on AI usage, faculty (97.62%) and students (86.51%) were the most common groups of focus. Like the data pertaining to AI applications, this data is likely explained by the guidance of AI usage in the classroom, being the most common theme among all documents.

### 4.2 Document screening and interrater reliability

The results of our interrater reliability analysis indicated low to moderate agreement per the extant literature, meaning that our determinations may not be reliable in triangulating findings across the reviewing team and future efforts (18, 19). However, Marzi also points out that some of the underlying algorithms of Krippendorf’s Alpha create the potential for sudden drops in the coefficients with comparatively small changes in interpretation; particularly with larger nominal data sets with low levels of expected disagreement like the one used in this study. Marzi et al. (19) write, “When the expected disagreement is low, even minimal variations can reduce Krippendorff’s Alpha to a minimal value.” Though this does not eliminate the possibility that the team inherently viewed the screening scheme differently, it provides a mathematical explanation for the lower coefficient.

### 4.3 TF-IDF analysis

TF-IDF analysis identified key terms across the 18 policies and their relative importance to the corpus of documents. Though there is no universal guidance on interpreting TF, IDF, or TF-IDF values, the results reveal patterns across all 18 documents that highlight the importance of certain topics. After cleaning and stemming, “use,” “student,” “tools,” “genai,” and “course” were the most common terms by word count, confirming that the policies as a corpus were primarily concerned with modalities of AI use in the context of the classroom. The individual IDF values align with this observation as well, with the first three terms appearing in every document, “genai” appearing in 17 of the 18 documents, and “course” appearing in 16 of the 18 documents. Given the homogeneity of topics covered across all documents in the corpus, the low values across the relevant coefficients are expected. However, these results triangulate with other analyses in this effort, as the most prominent words and their resultant TF, IDF, TF-IDF indicate that the policies are largely focused on applications of AI use in the classroom.

### 4.4 Thematic analyses

The results of the qualitative analyses illustrate both expected and dynamic elements of AI governance across the 18 institutions and their respective policies. Many of the policies, regardless of audience, primarily concern AI use regulation in the classroom. With the ongoing rapid embrace of generative AI tools across college campuses, embracing the field at large while avoiding generative AI misuse is a priority among institutions and academic organizations (22, 23). However, instead of solely focusing on preventing AI misuse directly, existing policies encourage faculty to adjust their course and curriculum design to facilitate effective and ethical generative AI use, with many offering resources to do so. The extant literature reflects this effort, with multiple studies providing theoretical frameworks for AI integration or recommendations for specific AI tools (24–27). With integration, additional language mandating transparency and practicality was often present, generally via faculty outlining their respective praxis on AI use in their syllabus.

However, the literature also reflects a degree of trepidation among faculty and students regarding AI integration, with descriptive studies showing both optimism and concern for a multitude of issues including institution-level support, long-term learning implications, potential for streamlined classroom processes (28–31). This trepidation is reflected in many of the policies as well, with many institutions lacking clear guidance on how to address AI misuse, and even less guidance on what AI misuse is outside of non-sanctioned use. As ubiquitous software programs continue to integrate AI into their processes, this lack of guidance on what qualifies as AI misuse could hinder effective integration.

Beyond regulation in the classroom, multiple themes pertaining to the ethical use of AI were present throughout many of the policies. Many of these, such as accountability and transparency, can be considered inherent risks of generative AI use, including accountability for AI-generated materials both in terms of product quality and academic integrity. However, topics such as privacy, harm-avoidance, and ownership were discussed at variable levels of detail and often lacked the same clarity of guidance when compared to other topics discussed. This is likely because these issues have not been resolved on a universal legal scale. Many states have enacted introductory laws regulating various dimensions of AI use, but these efforts represent the minority, and it remains unclear to what extent the federal government will regulate AI use (32). The inconsistency among states and current lack of federal oversight makes academic clarity difficult to achieve.

Discussions on the idea of ownership and intellectual property in the age of AI have permeated legal circles, with multiple works suggesting that these ideas core to academic processes be reconceptualized (33). As these discussions remain theoretical, academic responses have been equally inconclusive. This is reflected in the policies that mention risks to ownership of material, but nothing substantial in terms of safety and recourse beyond that. Work has begun in academic circles to answer these concerns, with select works suggesting frameworks for AI and IP policy creation (34). The intersection of privacy and AI ethics is similarly obfuscated, with the corpus of policies offering unclear guidance beyond caution or restriction on entering certain types of information into LLM’s. Without further consideration and development, this ambiguity could directly impact participation in academic exercises using AI. The notion of generative AI causing harm, regardless of how it manifests, is pressing and immediately pertinent to the field of public health and public health education at large. Select studies have sought to understand how to balance optimism and potential with the instances of LLM’s generating harmful or biased material in the health sciences (35). Despite the extent of discussion in and out of the academic arena, mention of how generative AI can cause harm in the corpus of policies is superficial or nonexistent.

Collectively, while issues of AI in the context of core academic function have received attention in the form of policy development and curricular design, broader ethical issues remain unresolved. This lack of clarity at both the macro and institutional level could explain the small number of mandated policies among ASPPH member schools. Executive bodies might be hesitant to issue mandated guidance to departments, faculty, staff, and students without further guidance and evidence from governing institutions and subject matter experts.

### 4.5 Limitations

There are several limitations in this study. First and foremost, the results and discussion reflect available AI use guidance until 3/1/2025. It is likely that some ASPPH member schools have since developed or enhanced their policies or guidelines to the point that they would have warranted inclusion into our final corpus of policies. As such, the results should be taken as a reflection of the state of AI guidance in ASPPH member schools during the document screening process, which lasted from approximately 10/2024 to 3/2025. Furthermore, it is important to note that many schools circulate AI policy internally and do not formally publish AI guidelines on their websites. Additionally, given the results of our interrater reliability analysis, it is possible that stakeholders outside of the research team might have different opinions on whether a certain school’s guidance should be considered a guideline or policy and whether it should have been considered for the subsequent analyses.

Additionally, it should be noted that the study analyzes publicly available institutional documents and therefore examines formal policy language rather than institutional practice or enforcement. Policies represent official statements of institutional expectations and governance structures but may not fully capture how AI use is implemented or regulated in practice. Consequently, the findings should be interpreted as an analysis of documented institutional guidance rather than direct evidence of operational behavior within universities.

## 5 Conclusion

This is the only known study that characterizes AI use guidance in schools and programs of public health. The findings of the collective analyses can be used to inform stakeholders of the current state of AI usage not only in schools and programs of public health but universities at large. Both the qualitative and quantitative results of said analyses indicate that the current environment of AI use governance is centered around student and faculty use in the classroom with an emphasis on effective integration, enforcement of academic integrity, and assurance of ethical use through multiple scopes (privacy, intellectual property rights, etc.). It is important to note that many schools circulate AI policy internally and do not formally publish AI guidelines on their websites.

There is a need to develop consistent, comprehensive policies on generative AI use across ASPPH member schools. However, existing program policies concerning AI rarely address it in context of community engagement. Furthermore, as associations like the ASPPH continue to emphasize the development of effective AI use regulation across its programs, the findings of this study can be used to identify gaps and areas for development in existing and forthcoming AI use governance. The intersection of interest, innovation, and capacity in higher education AI governance development has never been stronger. Although we demonstrate how current policies focus on academic integrity and ethical use, more schools are reviewing their programs to address AI integration in crucial career-building skills that will equip public health students for future career growth.

## Data Availability

The original contributions presented in the study are included in the article/supplementary material, further inquiries can be directed to the corresponding author.
