# Supplementary material for: Investigating the use of generative AI policies among ASPPH member schools and programs of public health
Source: Front Public Health. 2026 Apr 8;14:1796810. doi: 10.3389/fpubh.2026.1796810 (PMC13099806; doi:10.3389/fpubh.2026.1796810)
Supplement: Supplementary file 1 [file Table_1.docx]

| **AI Tool Name** | **Policy (n=18)** | **Guidelines (n=108)** | **Total (N = 126) (%)** |
| --- | --- | --- | --- |
| ChatGPT, ChatGPT3, ChatGPT4, Open AI's Chat GPT, Open AI GPT-4, GPT | 11 | 87 | 98 (77.78) |
| DALL-E, DALLE2 | 2 | 23 | 25 (19.84) |
| Google Bard | 2 | 23 | 25 (19.84) |
| Google Gemini | 2 | 18 | 20 (15.87) |
| Claude3, Claude.ai, Amazon Claude, Claude's Anthropic | 1 | 19 | 20 (15.87) |
| Microsoft Copilot, Copilot | 3 | 14 | 17 (13.49) |
| Midjourney | - | 12 | 12 (9.52) |
| Adobe Firefly | - | 11 | 11 (8.73) |
| Perplexity | 1 | 10 | 11 (8.73) |
| GPT0 | - | 7 | 7 (5.56) |
| Grammarly | 1 | 5 | 6 (4.76) |
| Turnitin | - | 5 | 5 (3.97) |
| Microsoft Bing | 1 | 2 | 3 (2.38) |
| GitHub Copilot | - | 2 | 2 (1.59) |
| All Others | 4 | 89 | 93 (73.81) |
| No specific tool mentioned | 4 | 7 | 11 (8.73) |
