# Supplementary material for: Investigating the use of generative AI policies among ASPPH member schools and programs of public health
Source: Front Public Health. 2026 Apr 8;14:1796810. doi: 10.3389/fpubh.2026.1796810 (PMC13099806; doi:10.3389/fpubh.2026.1796810)
Supplement: Supplementary file 2 [file Table_2.docx]

| **Applications** | **Policy (n=18)** | **Guidelines (n=108)** | **Total, (N = 126) (%)** |
| --- | --- | --- | --- |
| Teaching | 17 | 96 | 113 (89.68) |
| Learning | 15 | 89 | 104 (82.54) |
| Work-Related | 5 | 18 | 23 (18.25) |
| Research | 4 | 17 | 21 (16.67) |
| Content Creation | 3 | 3 | 6 (4.76) |
| Data Analysis/ Coding | - | 4 | 4 (3.17) |
| Editing /Text processing | - | 2 | 2 (1.59) |
| Others (Brainstorming, pre-writing) | - | 3 | 3 (2.38) |
