# Supplementary material for: Investigating the use of generative AI policies among ASPPH member schools and programs of public health
Source: Front Public Health. 2026 Apr 8;14:1796810. doi: 10.3389/fpubh.2026.1796810 (PMC13099806; doi:10.3389/fpubh.2026.1796810)
Supplement: Supplementary file 3 [file Table_3.docx]

| **Key Considerations** | **Policy (n=18)** | **Guidelines (n=108)** | **Total, (N = 126) (%)** |
| --- | --- | --- | --- |
| Reliability of AI tools | 2 | 5 | 7 (5.56) |
| Bias issues | - | 8 | 8 (6.35) |
| Lack of accuracy | 3 | 7 | 10 (7.94) |
| Copyright Violation | - | 3 | 3 (2.38) |
| Lack of Privacy | 1 | 3 | 4 (3.17) |
| Lack of confidentiality | 4 | 12 | 16 (12.7) |
| Faculty should communicate AI usage in their syllabus | 6 | 6 | 12 (9.52) |
| Faculty should encourage the use of AI | 7 | 61 | 68 (53.97) |
| Students should follow directions on AI usage | 15 | 109 | 124 (98.41) |
