# Supplementary material for: Investigating the use of generative AI policies among ASPPH member schools and programs of public health
Source: Front Public Health. 2026 Apr 8;14:1796810. doi: 10.3389/fpubh.2026.1796810 (PMC13099806; doi:10.3389/fpubh.2026.1796810)
Supplement: Supplementary file 4 [file Table_4.docx]

| **Applicability** | **Policy (n=18)** | **Guidelines (n=108)** | **Total, (N = 126) (%)** |
| --- | --- | --- | --- |
| Faculty | 18 | 105 | 123 (97.62) |
| Students | 14 | 95 | 109 (86.5) |
| Staff | 7 | 39 | 46 (36.5) |
| Researcher | 2 | 19 | 21 (16.67) |
| Others | - | 4 | 4 (3.17) |
